# Lingual primary tuberculosis mimicking malignancy

**DOI:** 10.1016/j.amsu.2021.102525

**Published:** 2021-06-20

**Authors:** Bahaa Razem, Sami El Hamid, Iro Salissou, Mohamed Raiteb, Faiçal Slimani

**Affiliations:** a Service de Stomatologie et Chirurgie Maxillo-faciale, Hôpital 20 Août, CHU Ibn Rochd, B.P, 2698, Casablanca, Morocco; b Faculté de Médecine et Pharmacie, Hassan II University of Casablanca, B.P 5696, Casablanca, Morocco

**Keywords:** Tuberculosis, Tongue, Fissure, Case report

## Abstract

**Introduction:**

The constant increase in the incidence of tuberculosis, as long as the emerging global resistance to antituberculous drugs warrants an increased awareness of the possibility of Mycobacterium Tuberculosis in persistent lesions of the oral cavity. Lingual tuberculosis is a rarely described entity of extra pulmonary tuberculosis. It usually presents as a non-healing chronic mucosal lesion that may mimic malignant lingual neoplasms.

**Case report:**

In the present paper, we report a rare presentation of lingual tuberculosis in a 36 years old woman, which was clinically suspected as an extensive malignant fissure of the tongue. The diagnosis was confirmed by tongue biopsy and the patient responded well to the antitubercular chemotherapy.

**Discussion:**

lingual tuberculosis is a rare clinical and pathological entity of extrapulmonary tuberculosis. Only a few cases have been reported around the world, and in each one of them, a unique clinical form has been described. This variability of clinical presentations can allude to several pathological conditions including malignancy.

**Conclusion:**

This case report is a documentation of a unique clinical and radiological presentation of lingual tuberculosis; it also highlights the importance of considering tuberculosis in the differential diagnosis of chronic tongue lesions.

## Highlights

• Tuberculosis is one of the leading causes of death worldwide.
• Lingual tuberculosis is a rarely described entity of extra pulmonary tuberculosis.
• Primary tuberculosis of the oral cavity is exceedingly rare.
• Chronic ulcer is the most common clinical form of lingual tuberculosis, in our case it was manifested by a fissure with extensive infiltration of the soft tissues.

## 1 Introduction

Tuberculosis (TB) is a chronic infectious disease caused by Mycobacterium tuberculosis and less frequently by ingesting unpasteurized cow's milk infected by Mycobacterium Bovis or by other atypical mycobacteria [1]. In its 2020 global tuberculosis report, The World Health Organization estimated that about 10.0 million people were infected with TB in 2019 [2]. In Morocco, a total of 30,636 cases of tuberculosis, all forms combined, were notified in 2015, i.e. an incidence of 89 cases per 100,000 inhabitants [3]. Extra-pulmonary tuberculosis accounts for 20% of tuberculosis infection cases, it could be either primary or secondary to a pulmonary location. In the head and neck area, the most common onset is cervical lymphadenitis, which accounts for 95% of ENT cases [4]. Tuberculosis of the tongue is a rarely described entity with a rate of 0.1% [5].

### 1.1 Case report

This case report has been registered on researchregistry.com under the reference: researchregistry6802.

Our work is a single case report and has been reported in line with the SCARE criteria [6].

We report the case of a 36-year-old patient, single, residing in Casablanca, who presented to the maxillofacial surgery department in March 2021 for a constriction of mouth opening progressively evolving over the past six months, following the extraction of the left mandibular third molar, which was constantly irritating the left side of the tongue. She had no history of cough, hemoptysis, fever or digestive disorders. The patient denied having any type of alcohol and tobacco consumption and any type of sexual relations.

**Fig. 1:**
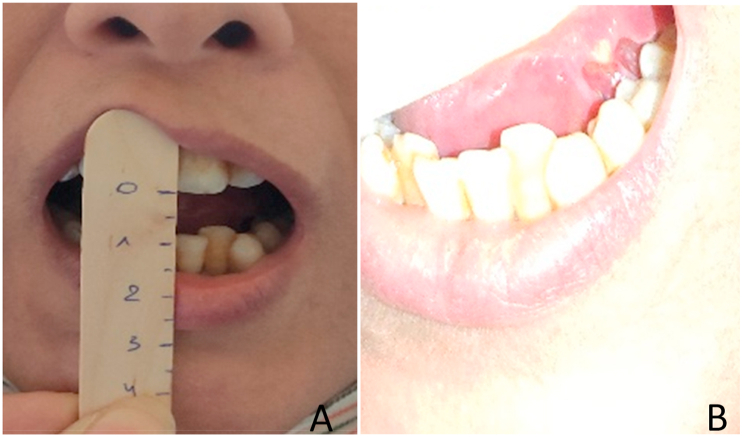
Photographs of the patient chowing the severe trismus (A) and the fissure of the undersurface of the tongue (B).

The clinical examination found a patient in a good general built with a performance status (PS) of 0 and a body mass index (BMI) of 21,3 Kg/m2. The oral cavity examination showed a tight trismus at 8 mm with limited lingual protrusion deviated to the left side. We also noted the presence of a 1 cm long fissure in the undersurface of the tongue (left side) and a regular induration of the retromolar trigone (Fig. 1 A and B), the base of both lesions was firm in consistency on digital palpation. Nasopharynx and oropharynx were clinically normal.

The remainder of the somatic examination was unremarkable, including no signs of pleural effusion, hepatomegaly, splenomegaly, ascites or palpable lymphadenopathy.

A panoramic radiograph showed no evidence of bone involvement. The laboratory examinations showed that complete blood count was within normal limits. Serologic tests for human immunodeficiency virus, viral hepatitis B and C and syphilis were all negative.

**Fig. 2:**
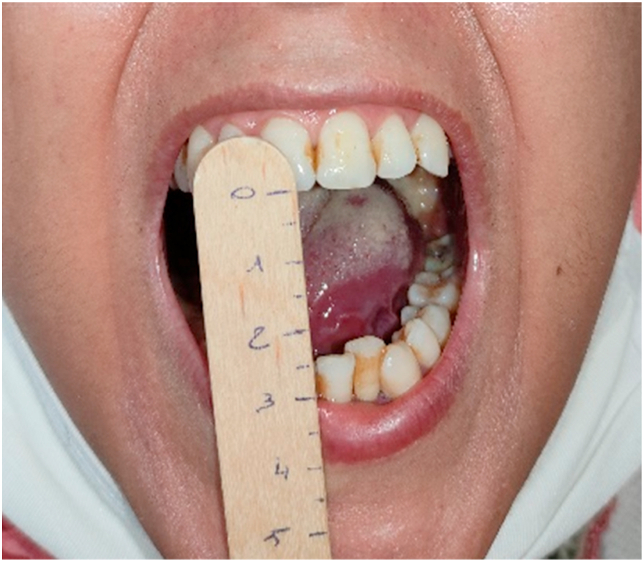
Photograph of the patient chowing her mouth opening after one month of treatment.

Faced with the repetitive irritation of the tongue, the short duration of evolution and the clinical manifestations of the patient in favor of a lingual lesion, infiltrating the base of the tongue, the retromolar trigone and the infratemporal fossa, a locally advanced carcinoma was mentioned first. A facial MRI was performed revealing the presence of a lesional process at the expense of the left side of the tongue, extended to the base, to the retromolar trigone and to the left parapharyngeal space, without dividing border with the medial pterygoid muscle. The surgical biopsy taken from the lingual fissure revealed clusters of epithelioid cells, caseating necrosis and numerous Langhans-type giant cells surrounded by a chronic inflammatory type of infiltrate. The tuberculin (Mantoux) test was positive, suggesting tubercular infection. Chest radiography revealed no abnormalities. Three sputum specimens were smear negative and culture negative. Antitubercular treatment was started with Isoniazid, Rifampicin, Pyrazinamide and Ethambutol for 2 months (2RHZE) and the patient was asked to continue with the first two drugs for the next 4 months (4RH). The three months evolution under treatment was very favorable with a gain of 8 mm on the mouth opening at the end of the 1st week and of 24 mm at the end of the 1st month and a total disappearance of the limitation of the lingual protrusion and the fissure at the end of the 2nd month (Fig. 2).

## 2 Discussion

Tuberculosis is one of the top ten leading causes of death worldwide [7]. Tuberculosis of the head and neck region can be a secondary site to a pulmonary location, or as in our case, a primary infection [9]. Oral TB is estimated to represent only 0.05–5% of total TB cases [10].

Primary form of tuberculous oral lesions is commonly encountered in children and adolescents. The secondary form is more frequent in adults and seniors mainly involving the tongue and the hard palate [8].

The scarcity of lingual TB is mostly due to the pH of the oral cavity, the intense blood supply, paucity of lymphoid nodes and to the physical effect of saliva allowing continuous cleansing of the oral mucosa [1,10,11].

Immunodeficiency is highly correlated with all forms of TB, it is estimated that in South Africa 30% of HIV-seropositive subjects have active TB [12]. On the other hand, in the case of immunocompetent patients, local conditions may allow the infection. Such patients often have a history of trauma or chronic inflammation or irritation favorising localization of mycobacterium: chronic tobacco use, traumatizing, dentures, poor oral hygiene or tooth extractions are the most described [10]. Oral tuberculosis after dental manipulation has been documented [1]. Furthermore, in areas where unpasteurized milk is consumed, M. bovis has been described to be responsible of alimentary contagion of the oral cavity [13].

Lingual tuberculosis is mostly described to be located in the lateral borders, the tip and the posterior third of the tongue, while ventral and anterior surface are relatively rare entities [5,11]. The usual presentation is an irregular, painful ulcer which can mimic trauma, or malignancy when slowly increasing in size. Swelling, tuberculoma, cold abscess, fissured and nodular lesions represent other mucosal manifestations [4,5,7,9,10,[14], [15], [16], [17]]. Table 1 resumes some of the reported lingual tuberculosis cases comparing them with our case.

**Table 1:** Examples of cases describing different locations and clinical presentations of lingual tuberculosis, with differential diagnosis and histological findings.

Study	Age	Gender	Location	Clinical presentation	Provisional diagnosis	Histological finding
Younes,al (2021) [14]	46	M	Lateral border	Cold abcess	–	Caseating granuloma with necrosis
Museedi,al (2020) [7]	48	M	Tip	Painless ulcerated mass	Squamous cell Carcinoma (SCC)	Caseating granuloma with necrosis
Kim,al (2019) [9]	57	M	Tip	Painful ulcer	Aphthous ulcer, traumatic ulcer, granulomatous diseases, infections.	acid-fast bacilli (AFB)
Estomba,al (2015) [4]	62	F	Base	Ulcer	–	necrotizing granulomatous inflammation with AFB
Yadav,al (2012) [10]	65	M	left side of anterior 2/3rd	Hemimacroglossia	submucosal carcinoma	granulomatous inflammation with AFB
Al-Rikabi,al (2011) [15]	42	M	Lateral border	Persistent ulcer	SCC	Caseating epithelioid and giant cells granuloma with AFB
Gupta,al (2007) [16]	25	M	Dorsal surface	Painful tuberculoma	–	Tuberculous granulomatous lesion
Das,al (2006) [17]	32	M	Lateral border	Painful ulcer	Malignancy	Necrotizing granuloma with giant cells
Our case (2021)	36	F	Ventral surface	Fissure and trismus	SCC	Granuloma with caseating necrosis

This variability of clinical presentations can allude to several pathological conditions. A variety of pathologies are to be mentioned: traumatic ulcer, aphtous stomatitis, syphilis, sarcoidosis, Wegener's granuloma, actinomycosis, parasitological and mycotic infections and of course squamous cell carcinoma are differential diagnosis to discuss [1,4,9].

This non specific presentation of tongue and oral TB results in laboratory confirmation being an essential requirement for diagnosis. Ziehl -Neelsen strain and culture in a histologic examination confirms the diagnosis [18]. The caseation pattern has been considered a more specific and sensitive typical histopathologic pattern of TB. However, noncaseating Langerhans granulomas, with or without necrosis, are also possible [7]. An investigation of a possible pulmonary involvement is mandatory.

Isonazid, Rifampicin, Pyrazinamide and Ethambutol, is the most common scheme of therapy [4]. The clinical lesions are described to improve after a few monts, effectiveness of chemotherapy appears to be due to the efficient vascularity of the tongue [5]. But even if lesions are improving relatively fast, a long chemotherapy is needed to achieve healing. The mean time described of therapy ranges from 6 to 15 months [19]. In our case, the patient will be receiving 6 months of treatment.

## 3 Conclusion

The recent outbreak of Coronavirus reminded us how easy it is for a microorganism infection to thrive even with all the efforts made to control it. Despite numerous advances in prevention, diagnosis and treatment, tuberculosis remains a major cause of death around the world, mostly in developing countries. Despite being a rare manifestation of tuberculosis, oral lesions should be included by maxillofacial practitioners in their differential diagnosis of any lesions in the oral cavity in general, especially for those not responding to conventional treatments. Histological examination plays a key role in establishing the diagnosis. This is especially important considering difficult clinical diagnosis because TB can mimic a variety of other conditions including squamous cell carcinoma. Lingual TB does not need surgical resection and prognosis is favorable after antituberculous treatment.

## Ethical approval

Our study is exempted from ethnical approval.

## Sources of funding

None.

## Author contribution

Bahaa RAZEM: Study concept, data collection, writing the paper and making the revision of the manuscript following the reviewer's instructions.

Sami EL HAMID: Study concept and writing the paper.

Iro SALISSOU: Revision of the manuscript following the reviewer's instructions.

Mohammed RAITEB: Revision of the manuscript following the reviewer's instructions.

Faiçal SLIMANI: reviewing and validating the manuscript's credibility.

## Registration of research studies

Name of the registry: research registry.

Unique Identifying number or registration ID: researchregistry6802.

Hyperlink to your specific registration (must be publicly accessible and will be checked): https://www.researchregistry.com/browse-the-registry#home/registrationdetails/609846d08b9f19001c0c7edc/

## Guarantor

RAZEM Bahaa.

## Consent

Written informed consent was obtained from the patient for publication of this case report and accompanying images. A copy of the written consent is available for review by the Editor-in-Chief of this journal on request.

## Declaration of competing interest

Authors of this article have no conflict or competing interests. All of the authors approved the final version of the manuscript.
